# Comparison of Supervised and Unsupervised Deep Learning Methods for Medical Image Synthesis between Computed Tomography and Magnetic Resonance Images

**DOI:** 10.1155/2020/5193707

**Published:** 2020-11-05

**Authors:** Yafen Li, Wen Li, Jing Xiong, Jun Xia, Yaoqin Xie

**Affiliations:** ^1^Institute of Biomedical and Health Engineering, Shenzhen Institute of Advanced Technology, Chinese Academy of Science, Shenzhen 518055, China; ^2^Shenzhen College of Advanced Technology, University of Chinese Academy of Science, Shenzhen 518055, China; ^3^Department of Radiology, Shenzhen Second People's Hospital, The First Affiliated Hospital of Shenzhen University, Shenzhen 518035, China

## Abstract

Cross-modality medical image synthesis between magnetic resonance (MR) images and computed tomography (CT) images has attracted increasing attention in many medical imaging area. Many deep learning methods have been used to generate pseudo-MR/CT images from counterpart modality images. In this study, we used U-Net and Cycle-Consistent Adversarial Networks (CycleGAN), which were typical networks of supervised and unsupervised deep learning methods, respectively, to transform MR/CT images to their counterpart modality. Experimental results show that synthetic images predicted by the proposed U-Net method got lower mean absolute error (MAE), higher structural similarity index (SSIM), and peak signal-to-noise ratio (PSNR) in both directions of CT/MR synthesis, especially in synthetic CT image generation. Though synthetic images by the U-Net method has less contrast information than those by the CycleGAN method, the pixel value profile tendency of the synthetic images by the U-Net method is closer to the ground truth images. This work demonstrated that supervised deep learning method outperforms unsupervised deep learning method in accuracy for medical tasks of MR/CT synthesis.

## 1. Introduction

Cross-modality medical image synthesis between magnetic resonance (MR) images and computed tomography (CT) images could benefit medical procedures in many ways. As a multiparameter imaging modality, magnetic resonance imaging (MRI) provides a wide range of image contrast mechanisms without ionizing radiation exposure, while CT images outperform MR images in acquisition time and resolution of bone structure. CT is also related with electron density which is critical for PET-CT attenuation correction and radiotherapy treatment planning [1]. Generating synthetic CT (sCT) images from MR images makes it possible to do MR-based attenuation correction in PET-MR system [2–6] and radiation dose calculation in MRI-guided radiotherapy planning [7–9]. Synthesizing MR images from CT images can enlarge the datasets for MR segmentation task and thus improve the accuracy of segmentation [10].

In recent years, there have been many efforts to work on medical image synthesis between MR and CT images. Among all these methods, deep learning method exhibited superior ability of learning a nonlinear mapping from one image domain to another image domain. It can be classified into two categories: supervised and unsupervised deep learning methods. Supervised deep learning methods required paired images for model training. In the MR/CT synthesis task, MR and CT images have to be well-registered at first and then used as inputs and corresponding labels for the neural network model to learn an end-to-end mapping. Nie et al. [11] used three-dimensional paired MR/CT image patches to train a three-layer fully convolutional network for estimating CT images from MR images. Other researchers [4, 5, 12–15] have trained deeper network for MR-based CT image prediction. However, as for medical image dataset, it is not that easy to get paired MR and CT images. It may take a long time span to collect patients who are scanned by both MR and CT scanners. Registration of certain accuracy between MR and CT images are also necessary to make paired MR-CT dataset.

Unsupervised deep learning methods enabled the possibility of using unpaired images for image-to-image translation [16–20]. It was first proposed for natural image synthesis and now has been implemented by many researchers for medical image synthesis [10, 21–24]. Chartsias et al. [10] demonstrate the application of CycleGAN in synthesizing cardiac MR images from CT images, using MR and CT images of different patients. Nie et al. [21] synthesized MR images from CT images with a deep convolutional adversarial network. Since there are plenty of unpaired medical images, the available datasets could be easily enlarged.

Unlike natural images, accuracy is highly emphasized in medical images. In this paper, we aim to compare the accuracy of supervised and unsupervised learning-based image synthesis methods for pseudo-MR/CT generation tasks. Two typical networks of U-Net [25] and CycleGAN [17] were introduced as representatives of supervised and unsupervised learning methods, respectively. Mean absolute error (MAE), structural similarity index (SSIM), and peak signal-to-noise ratio (PSNR) of the synthetic results were calculated to evaluate their performance quantitatively. More detailed comparisons and discussions about the advantage and disadvantage of these methods are included in Results and Discussion.

## 2. Materials and Methods

### 2.1. Neural Network Models

In our experiments of pseudo-MR/CT generation tasks, U-Net and CycleGAN were used as the typical representative network of supervised and unsupervised deep learning methods, respectively.

U-Net has made a great achievement in segmentation tasks [25–29]. The advantage of U-Net is that it could use very few images to make a good performance. In this study, we adapted U-Net to an end-to-end image synthesis task.

**Figure 1:**
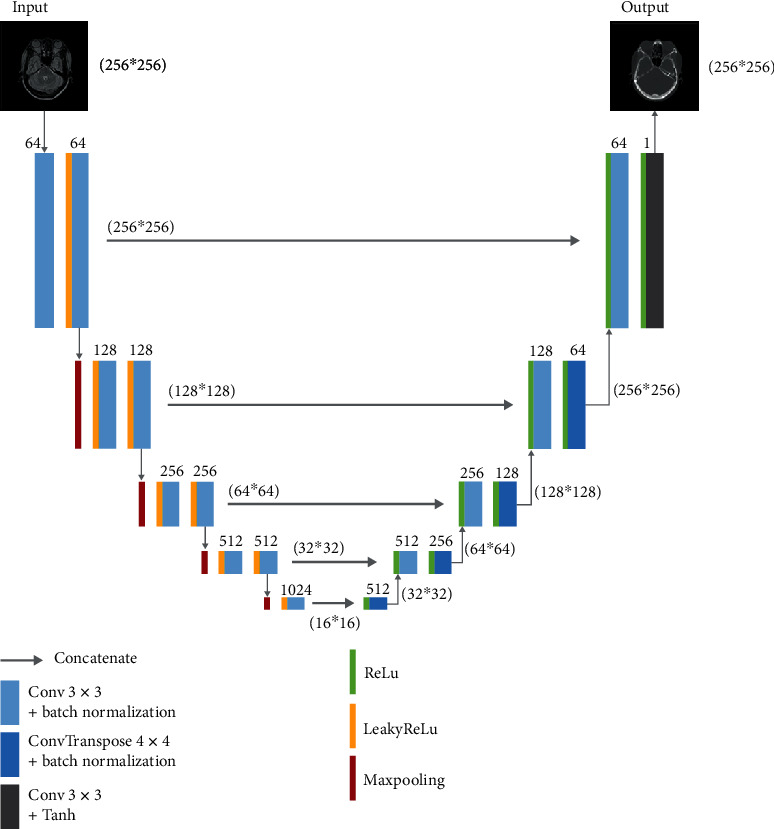
Architecture of proposed U-Net for image synthesis.

The basic architecture of U-Net consists of a contracting part to capture features and a symmetric expanding part to enable precise localization. As shown in Figure 1, we added LeakyReLU [30, 31] as activation operation before convolution operation in the contracting part of the network. Activation operation of LeakyReLU was replaced with ReLU [32] in the expanding part. Batch normalization [33] was introduced to U-Net to enable faster and more stable training. In Figure 1, the number of channels is denoted on top of each of the convolution operation, and the size of feature maps is signed in the parentheses.

In the medical image synthesis task, input image and its corresponding label were fed to the proposed U-Net to train and learn an end-to-end nonlinear mapping between them. Figure 1 illustrated the MR-to-CT synthesis using U-Net architecture, which takes MR images as input and CT images as label to train a synthetic CT generating model. On the contrary, when we use CT images as input and MR images as labels, U-Net could be trained as a synthetic MR-predicting model. The loss function used in the proposed U-Net is

(1) $$\begin{array}{c} L_{\text{U}­\text{Net}}={\text{E}}_{\text{x},\text{ y}\sim {\hat{P}}_{\text{data}}}\left[{\left\|f\left(x\right)-y\right\|}_{1}\right] \end{array}$$

CycleGAN [17] which is proposed by Zhu et al. could be seen as an updated version of generative adversarial networks (GAN) [16]. GAN methods can learn a nonlinear mapping from input image domain to target image domain by adversarial training. CycleGAN introduced the idea of cycle consistency to general GAN methods. Cycle consistency adds restriction that the generated pseudoimage in target domain should be able to be transformed back to the original input image.

**Figure 2:**
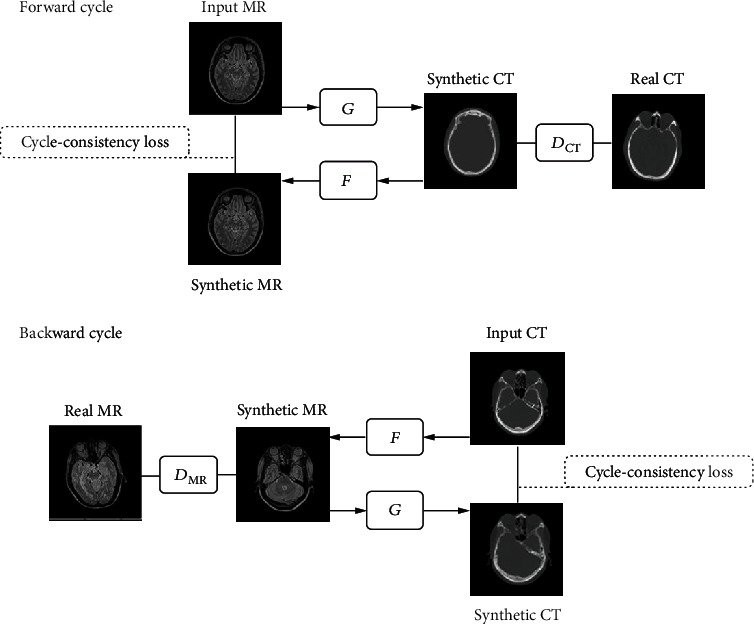
CycleGAN architecture for bidirection synthesis of MR and CT images. The forward cycle generated synthetic CT from input MR by *G* while *F* translate the synthetic CT back to the MR image domain. *D*_CT_ discriminate whether the generated images is real or fake CT. The backward cycle generated synthetic MR from input CT by *F* while *G* translate the synthetic MR back to the CT image domain. *D*_MR_ discriminate whether the generated images is real or fake MR. Two cycle-consistency loss was introduced to capture the intuition that the synthetic image should be translated back to the original image modality.

We used the CycleGAN architecture from Zhu et al. [17] for our medical image synthesis task. It takes unpaired MR and CT images as inputs to learn nonlinear mappings between these two image modalities. As illustrated in Figure 2, the CycleGAN architecture has two cycles, forward cycle and backward cycle. The forward cycle consists of three networks: two generative networks of *G* and *F* and one discriminator of *D*_CT_. The backward cycle uses the same generative networks of *F* and *G* and a counterpart discriminator of *D*_MR_.

In the forward cycle, network *G* was used to generate synthetic CT (sCT) from input MR images, while network *F* generated synthetic MR (sMR) from network *G*-generated sCT images. Network *D*_CT_ discriminates whether the generated sCT image is real CT or fake. The backward cycle works just the opposite way. Network *F* took CT images as input images and generated sMR; then, network *G* synthesized sCT from the *F*-generated sMR images. Network *D*_MR_ was used to distinguish whether the sMR image is real MR or fake.

The adversarial losses of CycleGAN are as follows:

(2) $$\begin{array}{c} L_{G\text{AN\_}G\text{\_M}{\text{R}}_{\text{to}}\text{CT}}={\text{E}}_{\text{CT}\sim P_{\text{data}}\left(\text{CT}\right)}\left[{\left\|\log\left(D_{\text{CT}}\left(\text{CT}\right)\right)\right\|}_{1}\right]+{\text{E}}_{\text{MR}\sim P_{\text{data}}\left(\text{MR}\right)}\left[{\left\|\log\left[1-\left(D_{\text{CT}}\left(G\left(\text{MR}\right)\right)\right)\right]\right\|}_{1}\right],\\ L_{\text{GAN\_}F\text{\_C}{\text{T}}_{\text{to}}\text{MR}}={\text{E}}_{\text{MR}\sim P_{\text{data}}\left(\text{MR}\right)}\left[{\left\|\log\left(D_{\text{MR}}\left(\text{MR}\right)\right)\right\|}_{1}\right]+{\text{E}}_{\text{CT}\sim P_{\text{data}}\left(\text{CT}\right)}\left[{\left\|\log\left[1-\left(D_{\text{MR}}\left(F\left(\text{CT}\right)\right)\right)\right]\right\|}_{1}\right]. \end{array}$$

The cycle-consistency loss consists of forward cycle loss *L*_forward_cyc_ and the backward cycle loss *L*_backward_cyc_. It is represented as follows:

(3) $$\begin{array}{c} L_{\text{forward\_cyc}}={\text{E}}_{\text{MR}\sim P_{\text{data}}\left(\text{MR}\right)}\left[{\left\|\left(F\left(G\left(\text{MR}\right)\right)-\text{MR}\right)\right\|}_{1}\right],\\ L_{\text{backward\_cyc}}={\text{E}}_{\text{CT}\sim P_{\text{data}}\left(\text{CT}\right)}\left[{\left\|\left(G\left(F\left(\text{CT}\right)\right)-\text{CT}\right)\right\|}_{1}\right],\\ L_{\text{Cycle}-\text{consistency}}=L_{\text{forward\_cyc}}+L_{\text{backward\_cyc}}. \end{array}$$

Then, we have the full objective as the below equation:

(4) $$\begin{array}{c} L_{\text{CycleGAN}}=L_{\text{GAN\_}G\text{\_M}{\text{R}}_{\text{to}}\text{CT}}+L_{\text{GAN\_}F\text{\_C}{\text{T}}_{\text{to}}\text{MR}}+\lambda L_{\text{Cycle}-\text{consistency},} \end{array}$$

where *λ* is the weight of the objectives of cycle consistency.

### 2.2. Cross-Modality MR/CT Image Synthesis and Evaluation

We used PyTorch to implement the proposed U-Net and CycleGAN. Both the networks were trained for bidirectional image synthesis, which includes learning a MR-to-CT model for generating synthetic CT images from MR images and a CT-to-MR model for generating synthetic MR images from CT images.

U-Net and CycleGAN used similar parameters for training nonlinear mapping models between MRI/CT images. Adam optimizer was adopted for both the networks. The batch size was set to 1. Both networks were trained for 200 epochs, with fixed learning rate for the first 100 epochs. The learning rate decreased linearly to 0 for the following 100 epochs.

Whole 2D slices of axial medical images with size of 256∗256 pixels were used as inputs. During the training process, the images would be padded to 286∗286 pixels and then random cropped to 256∗256 for data augmentation. While U-Net should utilize paired MR and CT datasets for training nonlinear mapping, CycleGAN can take use of unpaired MR and CT images as inputs for both the forward and backward cycles in training procedure. As for the CycleGAN method, we randomly shuffled the MR image input sequences and CT image input sequences in the paired datasets to make the input MR and CT slices unpaired. The MRI input sequence in unpaired datasets were not the same as that in paired datasets.

Three metrics were used to quantitatively characterize the accuracy of the prediction of synthetic images compared with the ground truth images. The mean absolute error (MAE) measures the discrepancies by voxels. Structural similarity index (SSIM) [34] quantifies the similarities in a whole image scale. Peak signal-noise-ratio (PSNR) assesses the quality of prediction.

These evaluation metrics are expressed as follows:

(5) $$\begin{array}{c} \text{MAE}=\frac{1}{H*W}\overset{H}{\underset{i=1}{\sum }}\overset{W}{\underset{j=1}{\sum }}\left|X\left(i,j\right)-Y\left(i,j\right)\right|,\\ \text{SSIM}=\left(2\mu_{x}\mu_{y}+c_{1}\right)\left(2\sigma_{xy}+c_{2}\right)/\left(\mu_{x}^{2}+\mu_{y}^{2}+c_{1}\right)\left(\sigma_{x}^{2}+\sigma_{y}^{2}+c_{2}\right),\\ \;c_{1}={\left(K_{1}L\right)}^{2},c_{2}={\left(K_{2}L\right)}^{2},\\ \text{PSNR}=10\;\log_{10}\left(L/\text{MSE}\right),\text{MSE}=\frac{1}{H*W}\overset{H}{\underset{i=1}{\sum }}\overset{W}{\underset{j=1}{\sum }}{\left(X\left(i,j\right)-Y\left(i,j\right)\right)}^{2}, \end{array}$$

where *H* and *W* are the height and width of the images, respectively. *X* is the ground truth images, and *Y* is the predicted synthetic images. *μ*_*x*_ and *μ*_*y*_ are the average values of ground truth images and synthetic images, respectively. *σ*_*x*_^2^ and *σ*_*y*_^2^ are the variance of ground truth images and synthetic images, respectively. *σ*_*xy*_ represents the covariance of ground truth images and synthetic images. *L* denotes the dynamic range of the voxel values. *c*_1_ and *c*_2_ are two variables to stabilize the division with a weak denominator. Here, we take *k*_1_ = 0.01 and *k*_2_ = 0.03 by default.

### 2.3. Dataset Preparing

The datasets contain 34 patients. Each patient has both T2-weighted MR images and CT images of the head region. We acquired T2-weighted MR images (TR: 2500 ms, TE: 123 ms, 1∗1∗1 mm^3^, 256∗256) on a 1.5 T Avanto scanner (Siemens). The CT images (120 kV, 330 mA, exposure time: 500 ms, 0.5∗0.5∗1 mm3, 512∗512) were acquired on SOMATOM Definition Flash (Siemens).

In this experiment, CT images were resampled to a size of 256∗256 (1∗1 mm^2^) by bicubic interpolation [35] to match the voxel size of MR images. Binary head masks were generated by the Otsu threshold method [36] for MR and CT images to remove unnecessary background information around the head region.

Since the head region is mainly a rigid construction of bone structure, we applied rigid registration to the MR and CT images to make paired MR/CT images for the proposed U-Net. CT images were set as a fixed volume. MR images were set as a moving volume to register with CT images by Elastix toolbox [37]. The paired datasets were randomly shuffled to make an unpaired dataset for CycleGAN.

In our medical image synthesis task, 28 patients with 4063 image pairs were randomly selected for model training. The remaining 6 patients with 846 image pairs were used for evaluation procedure.

## 3. Results and Discussion

**Figure 3:**
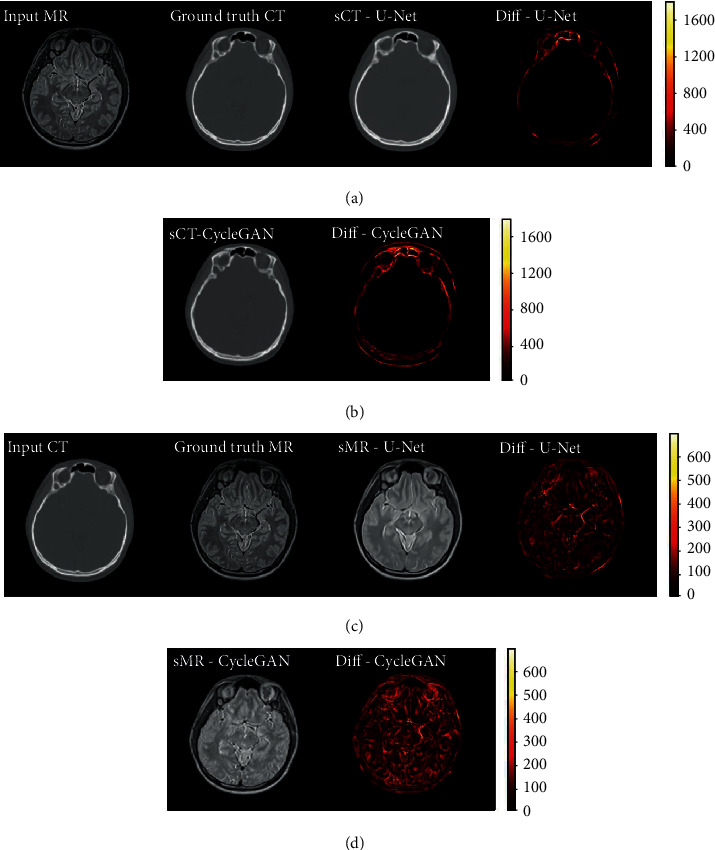
(a–d) From left to right: the 4 columns are input images, ground truth images, synthetic images, and the difference maps. sCT results generated by U-Net (a) and CycleGAN (b), respectively; sMR results by U-Net (c) and CycleGAN (d), respectively.

The results of synthetic MR and synthetic CT images generated by U-Net and CycleGAN and their ground truth are showed in Figure 3. The first column is the input images, and the second column is ground truth images. The third column showed the generated synthetic images predicted from input images by the two networks. The difference map between synthetic images and ground truth images was calculated and showed in the fourth column.

The first two rows in Figure 3 are sCT images synthesized by U-Net and CycleGAN, respectively. For the task of synthesizing CT images from MR images, the soft tissue area is translated from high contrast to low contrast. It could be seen from the difference map images that the soft tissue area of synthetic CT images by both networks is well-translated with little error. The translation error mainly occurred in the bone area. Their difference map demonstrates that the sCT by CycleGAN synthesized more error than sCT by U-Net in the bone areas.

The third and fourth rows in Figure 3 are sMR images generated by U-Net and CycleGAN, respectively. It could be seen that sMR by CycleGAN seems more realistic for it has more complex contrast information than sMR by U-Net. However, the difference map images illustrated that the CycleGAN method generated much more error than U-Net does. The abundant image contrast information in sMR by CycleGAN may be false and unnecessary.

In synthesizing CT tasks, the difference between synthetic images and ground truth mainly occurs in the bone area. But in synthesizing MR tasks, the error is evenly distributed in the whole head region. It means synthesizing high contrast images of MR from low contrast image domain of CT is tougher than its reverse synthesizing direction.

**Figure 4:**
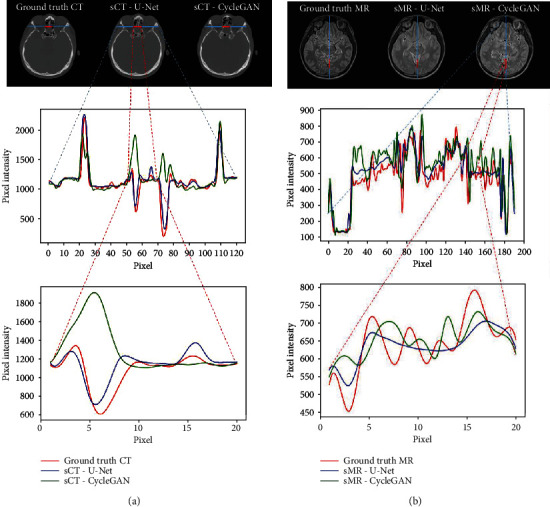
Comparison of 1D profiles of pixel intensity passing through the short red lines and long blue lines as indicated in the images: (a) 1D profile and its close-up marked by the horizontal lines in ground truth CT, U-Net sCT, and CycleGAN sCT images; (b) 1D profile and its close-up marked by the horizontal lines in ground truth MR, U-Net sMR, and CycleGAN sMR images.

To compare the image details, 1D profiles of pixel intensity were also plotted.  Figure 4 shows the 1D profiles passing through the short red lines and long blue lines as indicated in the corresponding images in the first row. The red line is overlapped with the blue lines. The 1D profile in the second row of Figure 4 demonstrates pixel intensities of the long blue lines. The 1D profiles in the third row are the pixel intensities of the short red lines of 20 pixels, which shows close-ups of part of the long blue lines' 1D profile.

In the profiles, the red curve indicates pixel intensities of ground truth CT or MR. The blue curve represented for U-Net and the green curve for CycleGAN. It could be clearly seen in Figure 4(a) that the blue curve is close to the red curve, while some of the peaks of the green curve deviated from the red curve to an opposite direction. It means that the tendency of 1D profiles in sCT by U-Net was closer to the ground truth CT, while the CycleGAN method tends to generate fake contrast information in sCT images.

The profile in Figure 4(b) shows that the blue curve vibrated less from the red curve. Some peaks of the green curve deviated more from the red curve. It could be seen in the close-up 1D profile that some peaks of the green curve are biased to the opposite from the red curve, while the tendency of the blue cure seems like a smoothened or flattened red curve. It means that the pixel value of sMR by U-Net was closer to the ground truth but may lack contrast details. The pixel value of sMR by CycleGAN exhibits more deviation from the ground truth along the profile whereas the tendency may be false or exaggerated.

**Figure 5:**
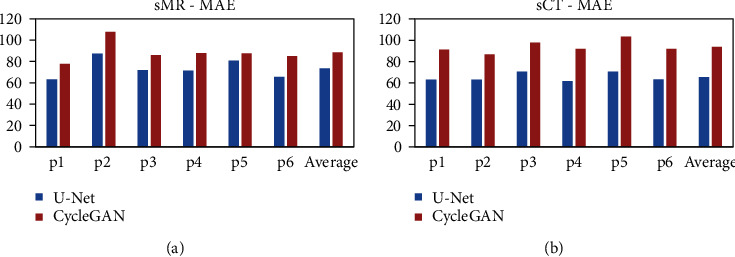
(a) MAE of sMR images for all the 6 patients in test set and their average value. (b) MAE of sCT images generated for all the 6 patients in test set and their average value. Both the blue columns denoted the U-Net method, and the red columns represented the CycleGAN method.

The quantitative metrics have been calculated for comparison.  Figure 5 shows the MAE of sCT and sMR for each of the 6 patients in the evaluation datasets and the average result. It is obvious that the U-Net method generated lower MAE either in sCT image generation or sMR image generation for all the patients. This also demonstrates the robust performance of the U-Net method in bidirection MR/CT image translation tasks.

Figures 5(a) and 5(b) show that the deviations of the MAE between the U-Net and CycleGAN method for sMR images of all the 6 patients are not as significant as those for sCT images. In Figure 3, the difference map of sMR indicated that the main predicted errors are evenly distributed in the whole head region, while the main error of sCT mainly occurs mainly in the bone structure. This could be interpreted that generating MR images of high soft tissue contrast from CT images of low soft tissue contrast is much complex than the inverse direction synthesis of generating CT from MR images.

Table 1 shows the overall statistics of three quantitative metrics for sCT by both the U-Net and CycleGAN methods. The SSIM values indicate that the sCT images by both methods have fairly high similarity with the ground truth CT images. The U-Net method outperformed the CycleGAN method with a much lower MAE of 65.36 HU, a higher SSIM of 0.972, and a higher PSNR of 28.84 dB. The average sCT MAE deviation between the two methods is nearly 30 HU.

Table 2 shows the overall statistics of three quantitative metrics for sMR images by the U-Net method and CycleGAN method. The U-Net method outperformed the CycleGAN method with a lower MAE of 73.43 HU, a higher SSIM of 0.946, and a higher PSNR of 32.35 dB.

The qualitative and quantitative results demonstrate that the proposed U-Net, a typical supervised learning method, outperforms CycleGAN, a representative advanced unsupervised learning method, in synthesis accuracy of medical image translation task. Since medical images highly value accuracy for the purpose of disease diagnosing, clinical treatment, and therapeutic effect evaluation, the supervised learning method is more recommended in medical practice.

Nevertheless, the success of supervised learning cannot do without well-registered image pairs. The performance of the trained model also depends on the registration accuracy of the paired images. Unlike natural images, paired medical images are not that easy to get. It would take a long time span to collect enough patients who need to be scanned for both MR and CT images at the same time. It is well-known that big amount of datasets could greatly improve the performance of the deep learning method. Though it outperforms the unsupervised learning method, the limit of dataset volume may constrain the further improvement of the supervised learning method in medical image synthesis tasks.

From the experiments discussed above, the image synthesis by using unsupervised learning methods still has a long way to go for practical application in clinic due to their relatively low accuracy. But still, the unsupervised learning method could benefit when there is lack of paired medical image datasets. The good news is that there are abundant easy-to-obtain retrospective unpaired MR and CT images for the unsupervised learning method to take advantage of. No registration is needed.

Our experiments show that when the same datasets were taken as inputs, the unsupervised learning method got inferior quality in the synthesis accuracy for medical image translation. But nonetheless, if the dataset is large enough, it could be expected that the performance of the unsupervised learning method would be improved to a certain acceptable extent in clinical practice.

## 4. Conclusions

Cross-modality medical image synthesis between MR and CT images could benefit a lot from the fast growing of deep learning methods. In this paper, we compared different deep learning-based image synthesis methods for pseudo-MR/CT generation, including the unsupervised learning method of CycleGAN and supervised learning methods of the proposed U-Net. Synthetic images produced by the CycleGAN method contain more but fake contrast information in the whole image scale. Though the proposed U-Net method blurred the generated pseudoimages, its pixel value profile tendency is basically close to the ground truth images. The quantitative results also indicate that the U-Net method outperformed the CycleGAN method, especially in synthesizing CT image task. As accuracy is highly demanded in medical procedures, we recommend the supervised method such as the proposed U-Net in cross-modality medical image synthesis at present clinical practice.

## Figures and Tables

**Table 1:** Quantitative evaluation results between ground truth CT images and sCT images: MAE, SSIM, and PSNR.

Model	MAE ± SD (HU)	SSIM ± SD	PSNR ± SD (dB)
U-Net	65.36 ± 4.08	0.972 ± 0.004	28.84 ± 0.57
CycleGAN	93.95 ± 5.89	0.955 ± 0.007	26.32 ± 0.55

**Table 2:** Quantitative evaluation results between ground truth MR images and sMR images: MAE, SSIM, and PSNR.

Model	MAE ± SD (HU)	SSIM ± SD	PSNR ± SD (dB)
U-Net	73.43 ± 9.16	0.946 ± 0.004	32.35 ± 0.78
CycleGAN	88.71 ± 10.04	0.924 ± 0.003	30.79 ± 0.73

## Data Availability

The datasets of MR and CT images used to support the findings in this study are restricted by the Medical Ethics Committee of Shenzhen Second People's Hospital in order to protect patient privacy.
